# Clinical Theragnostic Signature of Extracellular Vesicles in Traumatic Brain Injury (TBI)

**DOI:** 10.1021/acschemneuro.3c00386

**Published:** 2023-08-25

**Authors:** Anuvab Dey, Subhrojyoti Ghosh, Tiyasa Bhuniya, Madhurima Koley, Aishi Bera, Sudeepta Guha, Kashmira Chakraborty, Sathish Muthu, Sukhamoy Gorai, Rany Vorn, Chithravel Vadivalagan, Krishnan Anand

**Affiliations:** † Department of Biological Sciences and Biological Engineering, IIT Guwahati, North Guwahati, Assam 781039, India; ‡ Department of Biotechnology, IIT Madras, Chennai 600036, India; § Department of Biotechnology, NIT Durgapur, Mahatma Gandhi Rd, A-Zone, Durgapur, West Bengal 713209, India; ∥ Chemistry and Chemical Biology department, IIT(ISM), Dhanbad 826004, India; ⊥ Heritage Institute of Technology, Chowbaga, Anandapur, Kolkata 700107, India; # Department of Orthopaedics, Orthopaedic Research Group, Coimbatore 641045, Tamil Nadu, India; & Department of Biotechnology, Faculty of Engineering, Karpagam Academy of Higher Education, Coimbatore 641021, Tamil Nadu, India; % Rush University Medical Center, 1620 W Harrison St, Chicago, Illinois 60612, United States; ¶ School of Nursing and Medicine, Johns Hopkins University, Baltimore, Maryland 21287, United States; ▲ Department of Surgery, University of Michigan Medical Center, Ann Arbor, Michigan 48109, United States; ▽ Department of Chemical Pathology, School of Pathology, Faculty of Health Sciences, University of the Free State, Bloemfontein 9300, South Africa

**Keywords:** Traumatic brain injury, Extracellular vesicles, Biomarkers, Therapeutics

## Abstract

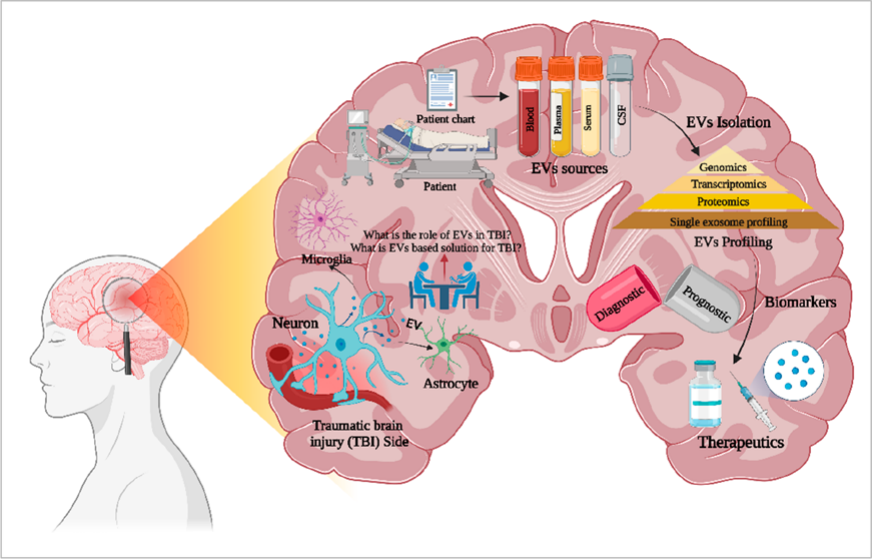

Traumatic brain injury (TBI) is a common cause of disability and fatality worldwide. Depending on the clinical presentation, it is a type of acquired brain damage that can be mild, moderate, or severe. The degree of patient’s discomfort, prognosis, therapeutic approach, survival rates, and recurrence can all be strongly impacted by an accurate diagnosis made early on. The Glasgow Coma Scale (GCS), along with neuroimaging (MRI (Magnetic Resonance Imaging) and CT scan), is a neurological assessment tools used to evaluate and categorize the severity of TBI based on the patient’s level of consciousness, eye opening, and motor response. Extracellular vesicles (EVs) are a growing domain, explaining neurological complications in a more detailed manner. EVs, in general, play a role in cellular communication. Its molecular signature such as DNA, RNA, protein, etc. contributes to the status (health or pathological stage) of the parental cell. Brain-derived EVs support more specific screening (diagnostic and prognostic) in TBI research. Therapeutic impact of EVs are more promising for aiding in TBI healing. It is nontoxic, biocompatible, and capable of crossing the blood–brain barrier (BBB) to transport therapeutic molecules. This review has highlighted the relationships between EVs and TBI theranostics, EVs and TBI-related clinical trials, and related research domain-associated challenges and solutions. This review motivates further exploration of associations between EVs and TBI and develops a better approach to TBI management.

## 1 Introduction

A change in brain function or brain damage caused by rapid brain movement is referred to as traumatic brain injury (TBI).^1^ TBI is thought to be a silent pandemic that causes a significant increase in global morbidity and mortality. This critical issue knows no bounds, impacting individuals of every age, gender, and race and wreaking havoc on their physical and financial well-being.^1,2^ Even though older people may experience fewer negative psychological effects from TBI since they have had more time to develop coping mechanisms,^3^ Maas et al. noted that the age group between 65 and 74 has the highest rate of TBI hospital admissions, followed by children and adolescents.^4^ The most recent recommendation from the National Academies of Sciences, Engineering, and Medicine for TBI reporting has modified the classification strategy. Now this process is reported directly based on a Glasgow Coma Scale scores.^5−7^ Additionally, increasing evidence suggests moderate-to-severe or recurrent mild TBI (mTBI) may increase Alzheimer’s disease risk^5,8,9^ and chronic traumatic encephalopathy.^10^ Mild TBI, such as concussions, accounts for the majority of TBIs. According to Carroll et al., between 20 and 50% of mTBI patients may experience persistent symptoms like depression and suicidal ideation.^11^ TBI raises the probability of neuropsychiatric and neurodegenerative illnesses.^12−14^ People with TBI have significantly higher rates of depression, suicidal ideation, and post-traumatic stress disorder than people without TBI.^13^

According to estimates, 27 to 69 million people worldwide suffer from TBI annually.^15^ According to multiple studies, the incidence of TBI ranges from 150 to 170 per 100,000 people in sub-Saharan Africa.^3^ The reported decrease in the incidence of TBI during COVID-19 lockdowns can be attributed to decreased mobility as well as decreased participation in sports and recreational activities.^16^ Development of advanced solutions, increased access to care, and encouragement of clinical research can all help prevent and manage TBI effectively.^3^ A system for acute care must be developed, and prehospital emergency treatment should be encouraged. Though the United States Department of Defense considered this issue 10–15 years ago, mTBI has recently been elevated as a research priority, and similar public health concerns must be raised in developing countries as well.^3,17^ Brain-specific TBI markers (GFAP (glial fibrillary acidic protein), NfL (neurofilament light), UCH-L1 (ubiquitin C-terminal hydrolase-L1), and total tau) have been extensively studied over the past decade, and are approved by FDA for diagnosis of mTBI.^18−20^

New TBI biomarkers, Identifed looks promising and therapeutically applicable. In the early stages of research, scientists delved into the intriguing world of fructose 1,6-diphosphate aldolase, glutamic pyruvic transaminase, GOT (glutamic oxaloacetate transaminase), LDH (lactate dehydrogenase), alpha-hydroxybutyric acid dehydrogenase, and malate transaminase enzymes, seeking to uncover their presence in the blood of individuals suffering from brain injury. This pioneering work opened the door to a new realm of scientific inquiry, ultimately leading to deep understanding and potential treatments for this devastating condition.^21,22^

**Figure 1:**
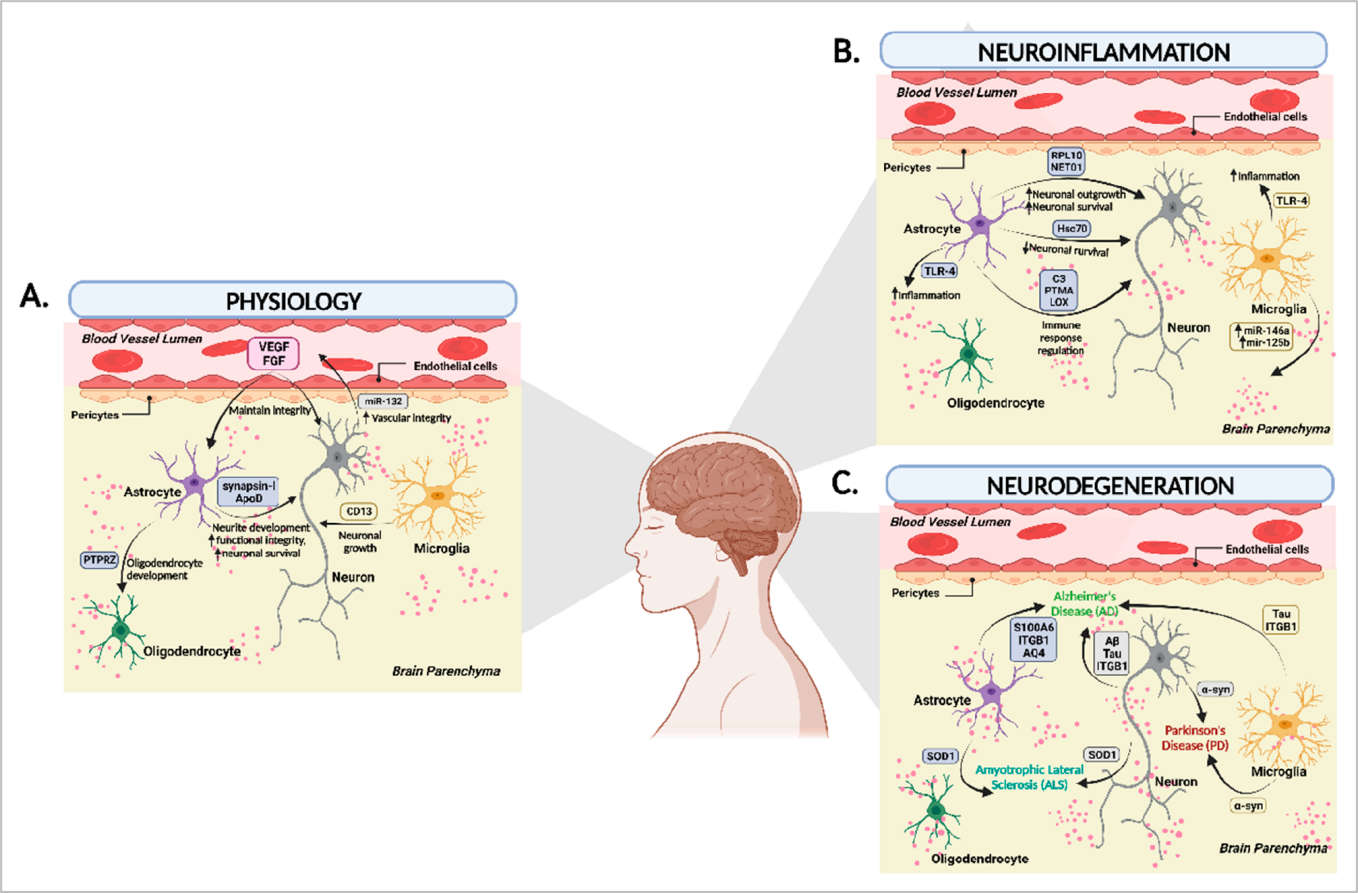
Interlink between EVs and brain function. (Created with BioRender.com).

In the cellular system, cells derived extracellular vesicles (EV) play the role of messenger (healthy or pathological condition).^23,24^ They are important for communicating between cells and transports dynamic bioactive compounds (DNA, RNA, proteins, etc.) (Figure 1).^25^ Nekludov and colleagues found that high levels of endothelial-derived exosomes in patients with severe TBI suggested vascular damage and microvascular thrombosis. The microvasculature of the brain is vulnerable to microparticles’ (MPs) (Microparticles are small membrane vesicles that are released from cells upon activation or during apoptosis) capacity to influence coagulation.^26^ The cerebral microvascular endothelium makes up the blood–brain barrier across transport. Excitotoxicity, abnormal cerebral blood flow, metabolic imbalance, and neuroinflammation are all caused by MP.^25^ EVs are thought to be present throughout the developing central nervous system (CNS) and are probably at least partially responsible for both normal and abnormal brain development during the embryonic and early fetal periods.^27^ Studies have shown that they are a desirable source of biomarkers because of their ability to enter the maternal circulation across the placental barrier and the fetal BBB.^28^

## 2 TBI-Associated Neurological Complications

When it comes to TBI, the damage commonly occurs due to external trauma, leading to profound brain dysfunction that can have a lasting impact on physical, cognitive, and emotional well-being.^29^ About 37% of injury-related deaths in trauma patients are attributable to TBI in Europe.^30^ The aftermath of a TBI can be fraught with a myriad of challenging neurological complications including seizures, dementia, Alzheimer’s disease, and injuries to cranial nerves. These complex and often debilitating conditions demand our attention and inspire us to pursue cutting-edge research and innovative treatments to improve outcomes for those affected by TBI.^29^ In addition, TBI individuals may also suffer several mental health issues, such as depression, post-traumatic stress disorder, generalized anxiety disorder, obsessive-compulsive disorder, and additional cognitive and behavioral sequels, which may significantly raise their morbidity.^29^ The frequency and severity of headaches can be significantly influenced by the severity of the injury.^11,28^ The genesis of headaches may be linked to a complex interplay between alterations in neuronal signaling, inflammation, and changes in the musculoskeletal system, all of which can be attributed to traumatic injury.^31^

By unraveling the intricate mechanisms underlying this debilitating condition, researchers can gain a deeper understanding of the underlying pathophysiology and pave the way for novel therapeutic interventions to alleviate the suffering of those afflicted by headaches.^11,28^ In their groundbreaking research, Riechers and colleagues shed light on the complex clinical picture of post-traumatic headaches, which often manifest as a hybrid headache disorder with features of both migrainous and tension-type headaches. Fortunately, they also discovered that a range of nonpharmacologic and pharmacologic strategies could be harnessed to treat these painful and often debilitating headaches, tailored to address the unique characteristics of the patients’ symptoms. This work represents a critical step forward in providing effective relief for those suffering from post-traumatic headaches.^28^

Corral et al.’s clinical survey found that 73% of patients had elevated intracranial pressure, with 51% developing intracranial hypertension and 56% developing low cerebral perfusion pressure at some point during their condition. About 28% of patients were found to be hypoxic upon admission, while 17% of patients were found to be hypotensive upon admission.^28^ Effective initial treatment of focal injuries, which can be fatal, requires prompt diagnosis and stabilization of patients with severe TBI.^32−34^ These factors collectively determine the patient’s prognosis, and their heterogeneity poses a significant challenge to clinical decision-making. Despite these obstacles, healthcare professionals and researchers are devoted to implementing innovative clinical strategies to improve outcomes for individuals with severe head injuries, offering hope for recovery.^32−35^

**Figure 2:**
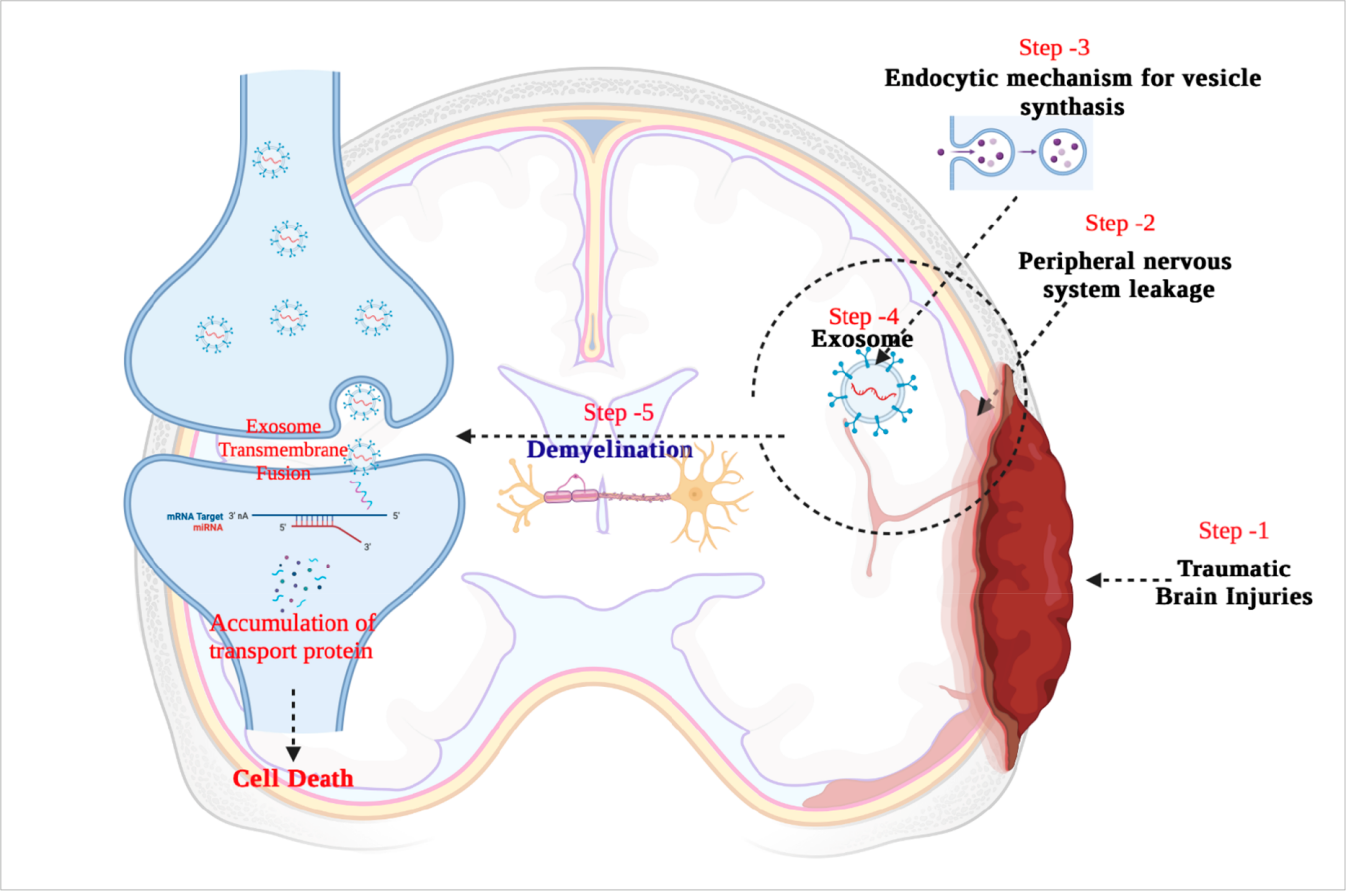
TBI secondary pathophysiology mechanism highlighting exosomal signal transduction via the apoptotic body endocytic pathway. Exosome secretory processes from apoptotic cell death after primary TBI cause secondary mechanisms of TBI and have a detrimental impact by impacting axon demyelination by transport protein accumulation and, ultimately, neuron cell death. Overall, signaling occurs via miRNAs and dysregulation of mRNA splicing mechanisms, resulting in unwanted protein accumulation in the axon and damage to the myelin sheath with demyelination processes, which result in different protein dysregulation and additional conditions that lead to: Ca^2+^ → mitochondria → ROS (reactive oxygen species) → caspases → DNA damage → cell death. (Created with BioRender.com).

In a study published in 2021, Hosomi and colleagues made a significant breakthrough in understanding gender differences in TBI-related mortality and morbidity, discovering that patient sex plays a crucial role in this complex phenomenon. Their research revealed that males were more likely to experience mortality from TBI than females, and this gender gap was most pronounced in both the youngest and oldest age groups. Furthermore, the study found that older men with TBI suffered more than the same-age female population.^36^ Their conclusion was also supported by extensive research using large samples from national registries.^36^ These findings have profound implications for clinicians and researchers alike, highlighting the urgent need to identify and address the underlying risk factors contributing to this disparity in outcomes for TBI patients.^36^ Gupte and colleagues proposed that such gender disparities may be attributed to various factors, including differences in TBI severity, patient age, race, physical condition, and the heterogeneous nature of TBI.^37^Figure 2 illustrates the signal transduction via an apoptotic body endocytic pathway from traumatic brain injury to neuronal cell death.

## 3 Extracellular Vesicles

EVs are cellular signaling molecule carriers originating from active cells.^23^ Although they are biologically present in healthy individuals, multiple studies demonstrate that pathological conditions like tissue hypoxia, oxidative stress, and cell activation increase their secretion.^38^ EVs are carriers of several bioactive cargos, such as DNA,^39^ RNA, proteins, etc.^25^ It is classified into exosomes, microvesicles (MVs), and apoptotic bodies.^40^ Biological fluids such as saliva, blood, plasma, serum, urine, and cerebrospinal fluid (CSF) sources of EVs. Exosomes are the byproducts of plasma-membrane-derived endosomes.^41,42^

**Figure 3:**
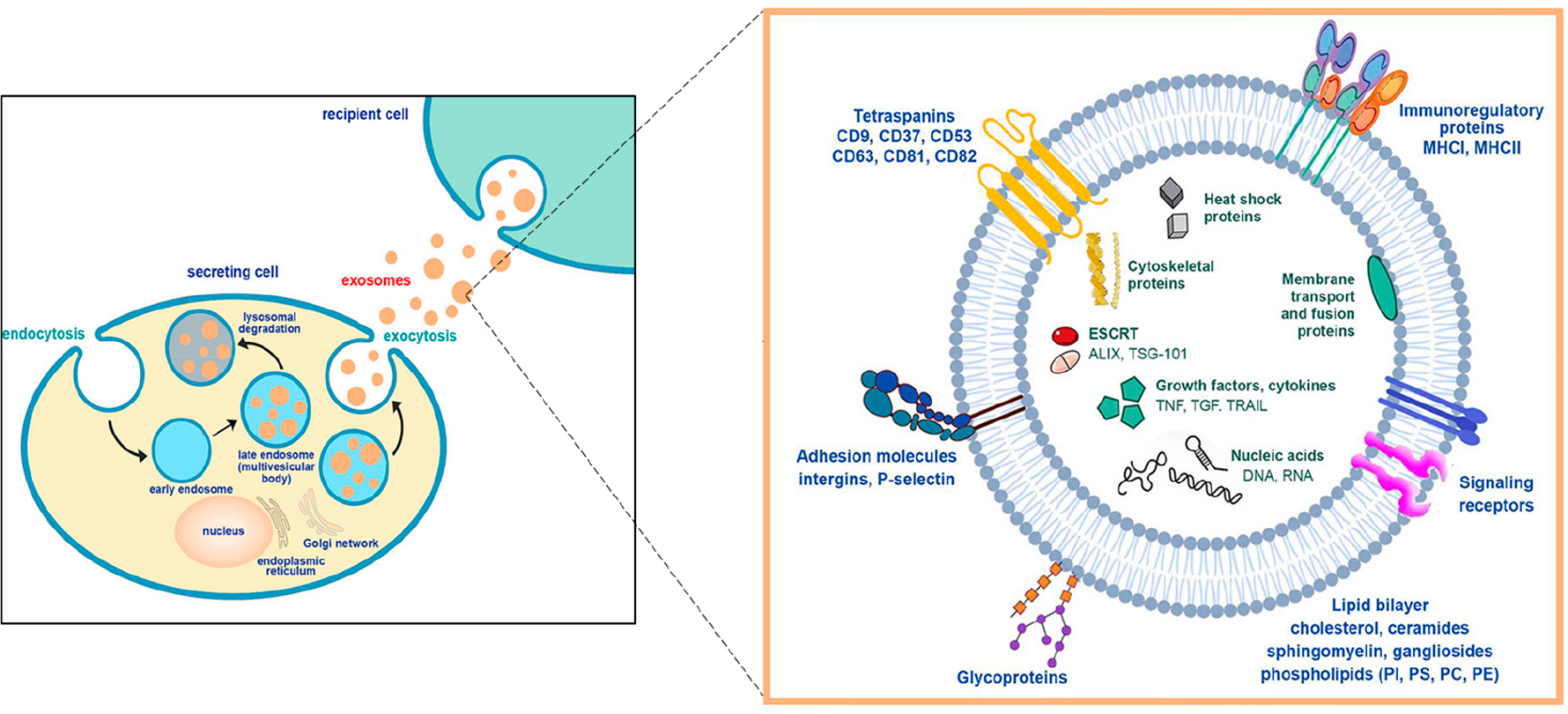
Exosome biogenesis and its molecular component. (Adapted with permission from ref (127). Copyright 2022 American Chemical Society).

Biogenesis of EVs is the combination of multiple molecules cascade. Processing of early endosomes (EEs) produces a subtype of endosomes carrying several membrane-bound intraluminal vesicles (ILVs) called multivesicular bodies (MVBs) (Figure 3).^43^ These MVBs subsequently fuse with the plasma membrane to release their contents outside the cells to form exosomes.^44^ There are two distinct mechanisms by which exosomes are produced: ESCRT (Endosomal Sorting Complexes Required for Transport)-dependent and ESCRT-independent.^44^ ILVs are produced by ESCRT using a sophisticated networking cascade^45−49^ involving four types of complexes such as ESCRT-0, ESCRT-I, ESCRT-II, and ESCRT-III.^50−52^ In the initial stages of the pathway, ESCRT-0 binds to Zinc Finger Domains (ZFDs) and Ubiquitin-Interacting Motifs (UIMs),^53^ present in the plasma membrane through its dimeric subunits like hepatocyte growth factor regulated tyrosine kinase substrate (HRS) and signal-transducing adaptor molecule 1/2 (STAM-1/2).^54^ This subsequently activates ESCRT-I and ESCRT-II, which facilitate cytoplasmic budding from the plasma membrane being guided by ESCRT-0, followed by mediation of cargo selection by ESCRT-II and ESCRT-III.^55^

The scientific rationale behind the ESCRT-independent pathway is not evident. It has been noted that ceramide-mediated membrane budding^57^ is linked to various cargo sorting and budding mechanisms. With their self-organizing ability and formation of a raft-like structure, membrane budding is enhanced during the biogenesis of exosomes.^52^ In various physiological conditions, distinct EVs types carry out unique functions. EVs are involved in the theranostic signature in TBI. Neuronal EVs are another possible source of EVs. Recent compelling data^44^ suggests that neuronal EVs act as mediators of neuronal plasticity, promoting neurogenesis and neurite outgrowth and thereby regulating and modifying neuroinflammation. Evaluating the amounts and temporal patterns of neuronal EVs in the blood and cerebrospinal fluid associated with TBI may provide crucial clues regarding the condition’s cause. Nekludov and colleagues found high levels of endothelial-derived MPs in patients with severe TBI, which is suggestive of vascular damage and microvascular thrombosis. The microvasculature of the human brain is susceptible to MPs’ capacity to influence coagulation.^26,56^ The cerebral microvascular endothelium makes up the BBB, which controls the diffusion and transport of solutes into the brain. Excitotoxicity, abnormal cerebral blood flow, metabolic imbalance, and neuroinflammation are all influenced by its altered or diminished permeability. These eventually lead to neuronal degeneration.^25^

## 4 Interrelation between TBI and EVs

EVs have emerged as crucial mediators of intercellular communication, playing a vital role in maintaining cellular homeostasis and regulating disease processes. Researchers can leverage EVs secreted by neuronal structures following neurological injury and disease to understand the biochemical molecular interlink between neurons and glial cells in health and disease. This multifaceted molecular signature of CNS-derived EVs makes them a promising tool for investigating the pathophysiology of neurological disorders (NDs).^58^ TBI has a postinflammatory immune response that causes local glia and recruited peripheral immune cells to become active and move toward the site of the damage.^59^ Current studies reveal that microglia-cell-derived EVs are associated with neuroinflammation.^60,61^ All brain cells, including neurons, astrocytes, microglia, and oligodendrocytes, have been shown to release EVs, which are essential for paracrine pathways mediating cell–cell communication in the brain.^44^ During TBI, within 24 h, the presence of microglia-derived EV-based pro-inflammatory molecules such as miRNA-155 and interleukin-1β are observed.^62,63^

**Figure 4:**
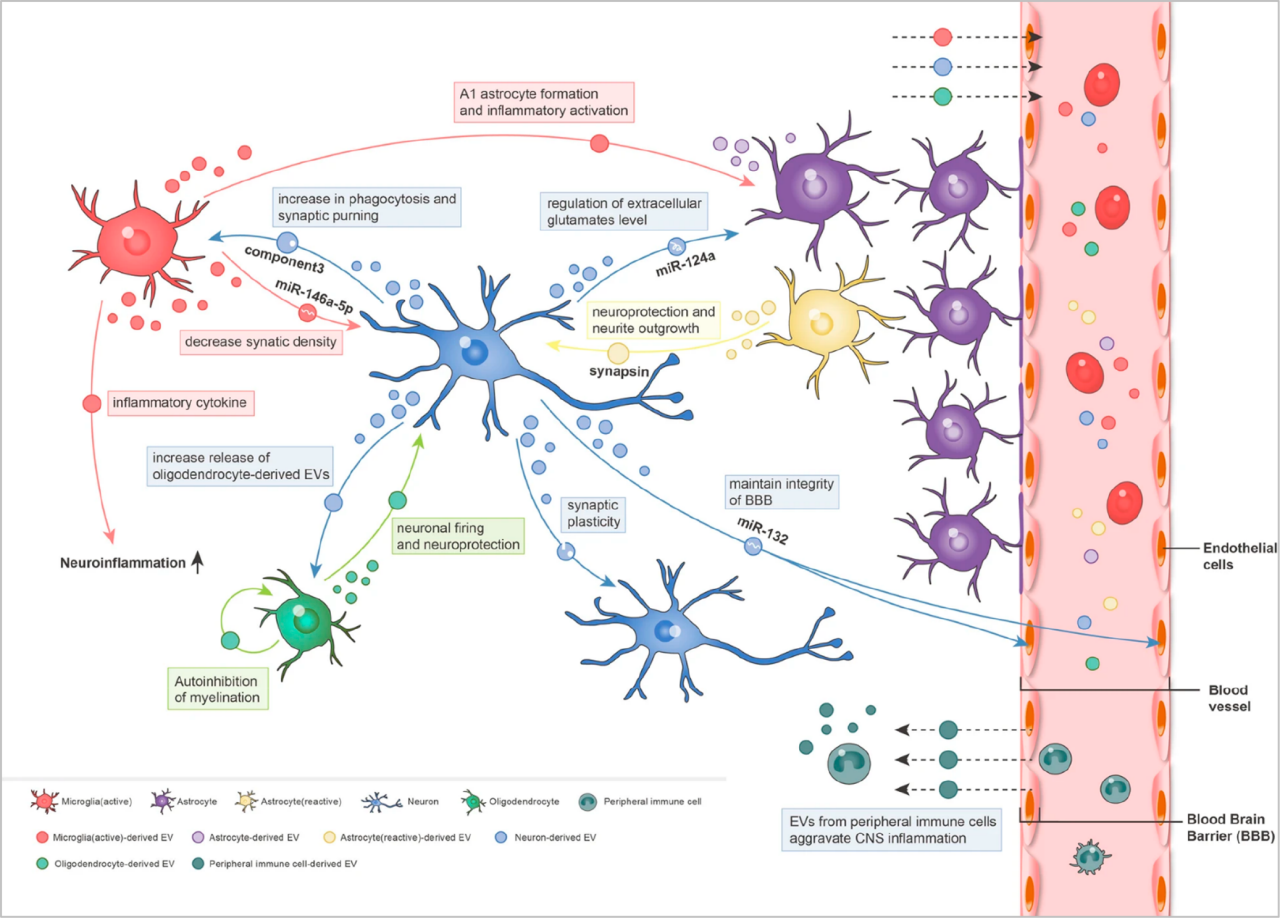
Interrelation of extracellular vesicles (EVs) and the central nervous system (CNS). (Adapted with permission from ref (66). Copyright licensed under a Creative Commons Attribution 4.0 International License, 2022, Stem Cell Research & Therapy, BMC).

Following an injury in a rodent TBI model, EVs were extracted, and the miRNA expression pattern was analyzed. miRNA-7, miRNA-21, and miRNA-146 expression were higher after damage whereas miR-212 expression declined, pointing to an enhancement loop of EV-induced neuroinflammation.^64,65^ These tiny messengers facilitate intercellular communication and have been found to carry key proteins implicated in the development of TBI and other NDs. Such findings emphasize the pivotal role of EVs in disease progression and provide new avenues for developing innovative diagnostic and therapeutic strategies.^40^ TBI has been linked to several NDs, according to certain studies. Because EVs can cross the BBB without causing immunogenicity, it surpasses the alternative synthetic drug delivery strategies aimed at affecting neuroinflammation, immunological responses, and sustained biodistribution (Figure 4).

Another critical stage in the development of TBI that EVs and their payload may influence is the change in the blood–brain barrier’s permeability. The blood–brain barrier may become more permeable in systemic inflammation, allowing EVs to penetrate the blood–brain barrier and trigger inflammatory processes in brain tissue.^6^ By altering the expression of vascular endothelial cadherin, neuron-derived exosomes with miR-132 were found to control blood–brain barrier permeability.^67^ Emerging evidence suggests that EVs’ internal molecular ingredients (such as miRNA) have a significant role in blood–brain communication during peripheral inflammation. Specifically, cerebrospinal fluid EVs transport pro-inflammatory signals and carry a group of miRNAs (miRNA-9, miRNA-1, miRNA-146, and miRNA-155) for brain cell communication. Such communication between choroid plexus epithelium cells and the central nervous system provides a unique mechanism by which the peripheral inflammatory status can be sensed and relayed to the brain.

These findings highlight the fascinating interplay between the immune and nervous systems and offer novel insights into the pathophysiology of inflammatory disorders.^68^ Due to their distinctive miRNA and protein fingerprints, EVs carry a signature of dynamic illnesses and disorders. In this occasion, EV-based TBI investigation requires more detailed molecular profiling of EVs of various neurological and non-neurological sources for the identification of biomarkers that will enable precise trauma assessment, clinical outcome prediction, and therapy optimization for specific individuals.

## 5 EV-Associated Biomarker of TBI

EV-based biomarkers of TBI remains a novel field that needs further exploration. These biomarkers are found in biological bodily fluids like blood, urine, saliva, and amniotic fluid as a part of systemic circulation (Table 1).^69,70^

**Table 1:** EV-Based TBI Biomarkers

Biomarker	EV source	Molecule	Clinical signature	References
Diagnostic	Blood, urine, saliva, amniotic fluid	Salivary S100B	Concentration increases in CNS	(71)
	CSF, Serum, Blood	MAPT	Elevated MAPT levels	(72)
	CSF	GFAP	Levels found to be at the peak after TBI	(73)
	CSF	Aβ42	Elevated accumulation	(73)
	CSF, blood	AQP4	Increased level	(74)
	CSF	miRNA-124–3p	Downregulated (Initial rise in the level occurs at three to 14 days following injury, but they start to decline after 42 days)	(73)
	Blood	miRNA-30d	Shows elevated levels of this miRNA	(73)
Prognostic	Acute TBI tissue plasma	miRNA-124	Upregulation holds the key to restoring lost neurological function	(58,75)
	CSF	miRNA-873a-5p	Increased level decreases brain edema by generating an anti-inflammatory effect.	(76)
	Blood	miRNA-146a	Increased production reduces the production of TRAF6 tLR-4 to NF-B pathway that leads to the decrease in the expression of downstream inflammatory proteins IL-1β, IL-6, and TNF-α.	(73)
	CSF, blood	miRNA-21	Upregulation is linked to better neurological prognosis	(67)
	CSF, blood	miRNA-141-3p	Upregulation observed in TBI further suggests the release of critical neuroinflammatory mediators.	(76)

The biggest fraction of Ca^2+^ binding proteins comprise Protein S100B, representing the S100 protein family. Astrocytes primarily generate S100B. The glial cells of the central nervous system have the largest concentration of S100B. Microtubule-associated protein tau (MAPT) is a hydrophilic intracellular protein with just a 10% concentration of α-helix and β-sheet secondary structure.^77^ In neurons, MAPT is persistently expressed and is greatly enriched in the axonal area. MAPT has significant immunoreactivity in the nonmyelinated axons of the cortical interneurons located in the gray matter of the human brain. MAPT release into the CSF and brain is a sign of neurotrauma and has been identified as a biomarker of axonal damage. Both moderate and severe TBI have been associated with elevated MAPT levels.^28,69,78,79^ When MAPT interacts with other motor proteins, the cytoskeletal network is assembled and stabilized.^77^

GFAP protein increases after a TBI, increasing the astroglia’s support, movement, form, and function.^9^ Also, GFAP may be used as a marker to distinguish MRI-positive TBI patients without CT findings from patients with negative MRI and CT.^80^ Even if these observations were from preparations of entire exosomes or EVs, it might be inferred that these signals originated from ADEs (astrocyte-derived exosomes) because GFAP is only generated in the cytoplasm of astrocytes.^73^ According to numerous studies, TBI is frequently associated with the marker amyloid beta 42 (Aβ42), and Aβ42 is enhanced in isolated exosomes or extracellular vesicles. Aβ accumulation in the soma and axon of neurons due to TBI may contribute to long-term neuronal damage.^73,81^

**Figure 5:**
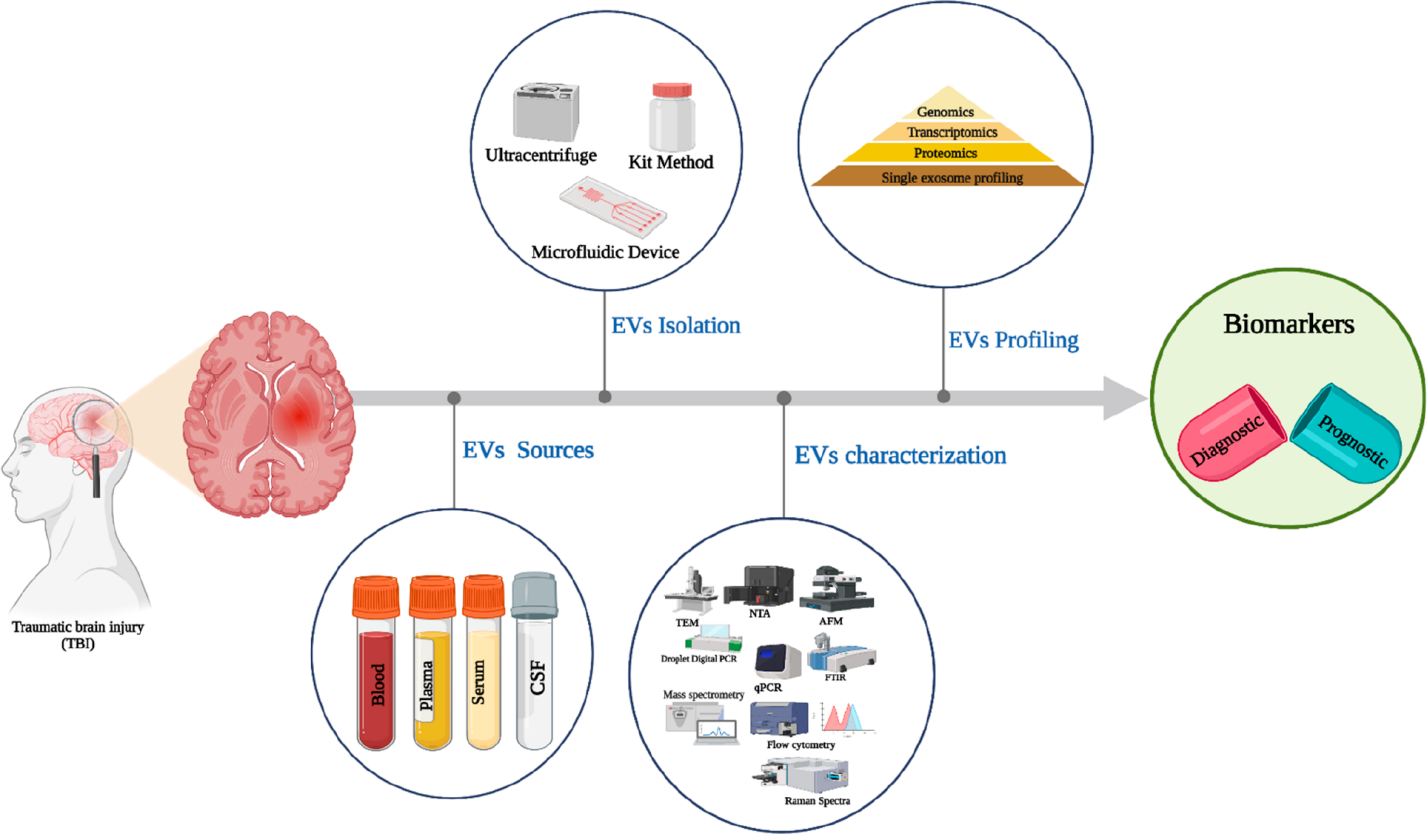
EV-based TBI biomarkers profiling. (Created with BioRender.com).

The dendrites of astrocytes contain a water channel protein called aquaporin-4 (AQP4) which may have a role in developing edema and neuroinflammatory processes. Both moderate and severe TBI had higher AQP4 exosome levels.^73,74,82^ The exosome miRNA investigation mentions that miRNA-124 downregulation is a sign of Alzheimer’s disease. The rise in these miRNA levels occurs at 3–14 days following injury, but they start to decline after 42 days.^73^ miRNA-146 in the EVs is significantly associated with brain inflammation complications. *Rhesus macaques* with virus encephalitis have higher levels of miRNA-146 in their EVs, and further investigation reasons this higher expression leads to IL-6-mediated brain damage. Since exosome miRNA-146 levels may help reduce brain inflammation, they may potentially enhance therapeutic results.^73^ Exosome-based probe development might aid in the detection of neuroinflammation through these candidate miRNA markers.^83^ Human astrocytes demonstrate elevated levels of miRNA-146 24 h after injury. It supports early injury detection.^73^ miRNA-21, despite being a tumor suppressor with anti-inflammatory property, exhibits noted upregulation following TBI and its levels in the brain has been linked to a better neurological prognosis.^67^Figure 5 explains the EV-associated TBI biomarker investigation approach.

## 6 TBI Profiling Approaches

Following brain trauma, immediate early genes, transcription factors, cytokines, and neurotrophic factors can all take part in the brain’s active and focused response, and they may even do so simultaneously, according to traditional methods for evaluating differential gene expression. New approaches for the thorough and simultaneous assessment of putative as well as new gene targets have been required due to the complexity and multiplicity of interconnected molecular pathways. Recent developments in DNA microarray technology have made it possible to simultaneously evaluate hundreds of genes and generate enormous amounts of biological data that are pertinent to CNS injury.^84^ Different biomarkers have been discovered and analyzed using a variety of metabolomics approaches. The majority of approaches rely on mass spectrometry (MS), which is frequently paired with chromatographic separation methods like gas or liquid chromatography (GC or LC). The use of proton nuclear magnetic resonance (^1^H-NMR) is also very common. The relative ease of sample preparation is a benefit of NMR over MS-based techniques.^85^

Changes in lipid profile patterns have been observed in TBI.^86^ Cardiolipins were found to be down-regulated in the perilesional region of an adult rat CCI (chronic construction injury) using MALDI-MSI (Matrix-assisted laser desorption/ionization–Mass spectrometry imaging).^87^ Further, its reduced levels lead to functional disorders of the brain.^84^ During brain damage, slow activation of phospholipases leads to primary lateral sclerosis.^88^ Recent lipid profiling on 18 dissimilar mouse tissues has shown that several sphingolipid subtypes are substantially abundant in the brain while being rather rare in other tissues. Sphingomyelin subtypes SM 36:1 and 36:2 concentrations in the brain are 20–90 times higher than in the plasma. These advantageous biochemical characteristics of sphingolipids would enable their early identification in circulating plasma following cerebral injury in a manner that is consistent with the severity of the lesion.^89,87,13,88^

Using several TBI models, many microRNAs were also identified in the brains of wounded animals. Some of these researchers have also looked at the potential pathobiology of the tissue-specific microRNAs that exhibit variable expression.^90^ The development of an effective approach for accurately stratifying patients based on their progression of disease remains a significant challenge in clinical practice. To address this issue, a novel method, termed “Dynamic Profiling,” was devised to enhance patient stratification. This approach involved the use of spectral Laplacian and Hartigan’s k-means algorithm to group patients into disjoint clusters at different phases of their illness trajectory. The initial grouping was based on the Glasgow Coma Scale (GCS) score, followed by clustering based on clinical and demographic data. Finally, sequential clustering was performed based on the levels of various inflammatory mediators over time. Overall, the proposed method may serve as a powerful tool for achieving more precise patient stratification in clinical settings.^91^ Brain network changes in patients with traumatic brain injury have been successfully described using graph theoretical analysis of the structural connectome. However, in the TBI population, neuropathology heterogeneity is a well-known problem, making it difficult to compare groups of patients with controls because of within-group variability.^92−94^

## 7 Therapeutic EVs for TBI

**Figure 6:**
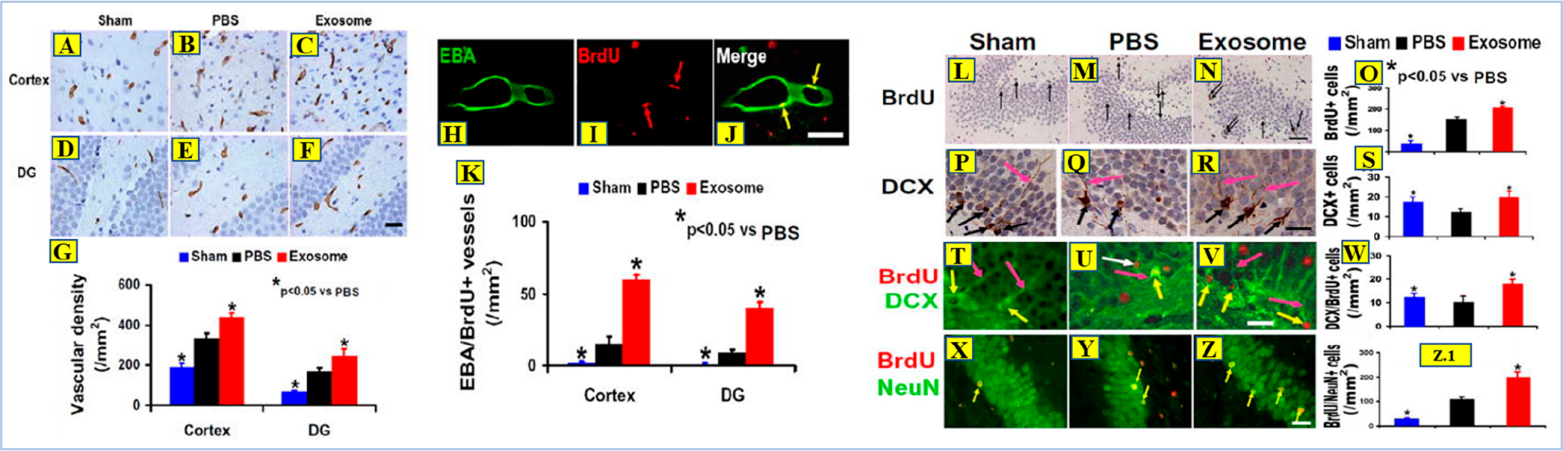
Therapeutic activity of mesenchymal stem cell-derived exosomes in TBI (treatment with exosomes derived from MSCs (mesenchymal stem cells)) significantly increases brain vascular density and angiogenesis in rats after TBI. EBA (endothelial barrier antigen) staining was performed for detection of mature vasculature at day 35 after TBI in the lesion boundary zone and dentate gyrus (DG) of the sham group (A and D), PBS-treated group (B and E), and exosome-treated group (C and F). Double staining for EBA (H, green) and BrdU (5-bromo-2′-deoxyuridine) (I, red arrows) to identify newly formed mature vessels (J, yellow arrows) in the brain at day 35 after TBI. Treatment with exosomes derived from MSCs significantly increased cell proliferation and neurogenesis in the DG of rats sacrificed at day 35 after TBI. BrdU staining for cell proliferation (L–Q, black arrows). DCX (doublecortin) staining for immature neurons (P–S, black arrows for DCX+ cells, and pink arrows for dendrites). Double staining with BrdU (red)/DCX (green) for newborn immature neurons is indicated by yellow arrows (T–W, pink arrows for dendrites). BrdU (red)/NeuN (green) for newborn mature neurons (X–Z, yellow arrows) are also labeled. (Adapted with permission from ref (100). Copyright licensed under a Creative Commons Public Domain Mark 1.0., 2015, J Neurosurg.).

EVs subpopulation exosomes are a potential therapeutic tool.^95,96^ They can connect with target neurons and glia even in the deepest parts of the brain and easily cross the blood–brain barrier.^97^ EVs have been identified, described, and specifically designed to promote positive outcomes in circumstances including disease and brain injury.^73^ EVs generated from neural and mesenchymal stem cells have demonstrated potential in treating brain dysfunction following disease or damage (Figure 6). Such characteristics of EVs formed from stem cells are crucial for clinical applications since EV therapy can mitigate several risks generally connected to cell therapy.^73,94,98,99^

Astrocytes create scars around TBI sites that prevent axon regrowth.^75^ By conducting experiments, Hira et al. observed that signaling element 3A prevents astrocyte activation and glial scar formation, and the regulation of those key components accelerates neural function recovery after ischemia, promoting axon growth, upregulating prostaglandin D2 synthase expression, and glial scar prevention.^101^

EVs miRNA is a major therapeutic molecular ingredient for several diseases. Gayen et al. observed that EV-derived miRNA-141 is a neuroinflammatory mediator in TBI and may be an important TBI biomarker. EVs miRNA-73 promotes the M2 population to microglia conversation during TBI. It also works as an anti-inflammatory signaling molecule.^4^ In the mouse model, EVs miRNA-873 cargo is associated with anti-inflammation and plays a role in TBI treatment.^76^ In acute TBI tissue, miRNA-124 produced by microglial EVs encourages M1 microglia to differentiate into M2 microglia, which dampens the neuroinflammatory response and neuron regeneration.^58,75,102,103^ Exciting research by Xie et al. reported that microglia-derived EVs miRNA-124 supports functional healing after TBI. New research has uncovered a potential breakthrough in TBI with the utilization of bone marrow mesenchymal stem cell-derived extracellular vesicles (BMSC-EVs) to transform microglia into an anti-inflammatory (M2 type) phenotype.^104^ This transformation includes the inhibition of pro-inflammatory cytokines IL-1, IL-6, and TNF- while promoting the production of the anti-inflammatory cytokines IL-10 and TGF-β.

Additionally, Sharma et al. found that miRNA-146a plays a crucial role in this process by reducing the production of TRAF6, a molecule that connects TLR-4 to the NF-κB (nuclear factor kappa B) pathway, leading to a decrease in the expression of downstream inflammatory proteins IL-1β, IL-6, and TNF-α, and ultimately the suppression of NF-B transcriptional activity. These findings could pave the way for a ground-breaking new method of treating TBI.^105^ So, this can be used as a possible therapy for TBI treatment.^14,106−109^ EVs derived from human umbilical cord blood endothelial colony-forming cells suppress PTEN expression. Deletion of PTEN causes Akt to change into p-Akt, which limits the activation of downstream apoptotic signaling pathways. This helps in the management of TBI by preventing the death of nerve cells by apoptosis and can act as a possible therapy for treating TBI.^110^

Overall, the study of EVs and their molecular cargo represents a rapidly growing field with significant potential for improving precision medicine in therapy. By providing noninvasive diagnosis, monitoring of disease progression and treatment response, and personalized treatment recommendations, EV biomarkers have the potential to revolutionize the field of medicine and improve patient outcomes.^111,112^

## 8 Future Prospective

**Figure 7:**
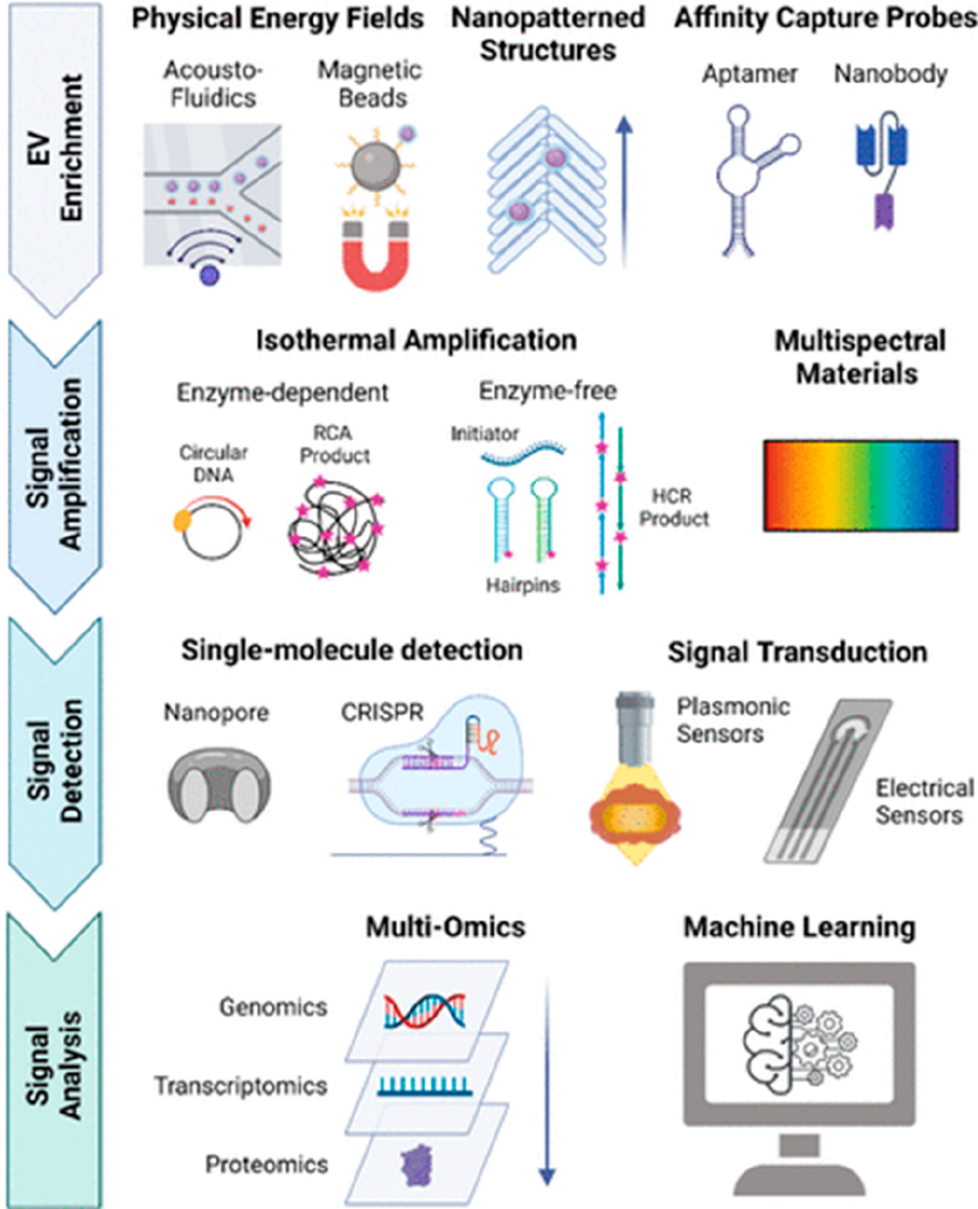
Single EV profiling approach. (Adapted with permission from ref (115). Copyright 2022 American Chemical Society).

EVs based TBI research faces several challenges such as EVs isolation related standard protocol and heterogeneity.^111^ Heterogeneity of EVs in TBI related to EVs size, shape, and cargo content diversity, sources of EVs (health cell or pathological complicated cells) .^112^ This complicates decoding via a single exosome profiling approach.^113^ A single exosome profiling approach involves advanced EVs isolation,^42,114^ multiomics profiling of EVs cargos,^115^ and combination machine learning (Figure 7).^73,115^ The next level of complexity lies in exosome toxicity. Exosome toxicity needs more clear scientific investigation for affordable and efficient exosome-based therapeutic approach development for TBI.^50,114−117^ Therefore, it is important to develop standardized methods for EVs isolation, purification, and analysis to ensure the reproducibility and comparability of results across different studies.^111^

The use of machine learning in analyzing EVs cargo data has shown promising results in developing predictive models for disease diagnosis and treatment response. These models can integrate multiple types of omics data, such as transcriptomics, proteomics, and metabolomics, to comprehensively understand disease and treatment response. By identifying patterns and associations between different variables, machine learning algorithms can provide personalized recommendations for treatment based on individual patient characteristics.^116,117^

The current research suggests mild TBIs may increase the risk of neurodegenerative diseases later in life if not given sufficient time to recover.^118^ These repetitive injuries are common in athletes and military personnel and remain under-reported. Thus, it is important to identify objective markers that outperform the standard assessments (such as quantitative assessments of symptoms, cognition, vestibule-ocular function, and dynamic balance) with the help of exosomal biomarkers.^119,120^ These expanded testing paradigms will benefit these communities and increase their safety. Exosomal biomarkers may provide valuable insights into brain-specific events after injury. They could be used as a “liquid biopsy” to determine what happens in the brain after an injury.^121^ Further research can provide insights into the long-term impact of these injuries, concerning Alzheimer’s disease or chronic traumatic encephalopathy with aging. By implementing these expanded testing protocols and conducting further research, we can improve the long-term health outcomes for individuals who have experienced minor TBIs.^122,123^ EV research requires an interdisciplinary research ecosystem,^52,124,125,126^ and we hope this approach supports us in developing a better solution for TBI.

## 9 Conclusion

EVs represent a promising avenue for developing a clinical theranostic signature in TBI. These tiny messengers play a crucial role in intercellular communication and have been found to carry a diverse range of cargo, including proteins, RNA, DNA, and metabolites. Recent research has revealed the EVs are the potential biomarkers for TBI diagnosis and prognosis. Moreover, the multifaceted molecular signature of EVs can offer insights into the pathophysiology of TBI and facilitate the development of innovative therapeutic strategies. However, further research is needed to explore the intricate mechanisms underlying EV-mediated intercellular communication in TBI and determine the clinical utility of EVs as biomarkers and therapeutic agents. Nonetheless, evidence suggests that EVs hold tremendous promise as clinical theranostic tools in TBI and other neurological disorders.
